# A Methodology for the Development of RESTful Semantic Web Services for Gene Expression Analysis

**DOI:** 10.1371/journal.pone.0134011

**Published:** 2015-07-24

**Authors:** Gabriela D. A. Guardia, Luís Ferreira Pires, Ricardo Z. N. Vêncio, Kelen C. R. Malmegrim, Cléver R. G. de Farias

**Affiliations:** 1 Department of Computer Science and Mathematics—Faculty of Philosophy, Sciences and Letters of Ribeirão Preto (FFCLRP)—University of São Paulo (USP), Ribeirão Preto, Brazil; 2 Faculty of Electrical Engineering, Mathematics and Computer Science—University of Twente, Enschede, the Netherlands; 3 Department of Clinical, Toxicological and Bromatological Analysis—Faculty of Pharmaceutical Sciences of Ribeirão Preto—University of São Paulo (USP), Ribeirão Preto, Brazil; Icahn School of Medicine at Mount Sinai, UNITED STATES

## Abstract

Gene expression studies are generally performed through multi-step analysis processes, which require the integrated use of a number of analysis tools. In order to facilitate tool/data integration, an increasing number of analysis tools have been developed as or adapted to semantic web services. In recent years, some approaches have been defined for the development and semantic annotation of web services created from legacy software tools, but these approaches still present many limitations. In addition, to the best of our knowledge, no suitable approach has been defined for the functional genomics domain. Therefore, this paper aims at defining an integrated methodology for the implementation of RESTful semantic web services created from gene expression analysis tools and the semantic annotation of such services. We have applied our methodology to the development of a number of services to support the analysis of different types of gene expression data, including microarray and RNASeq. All developed services are publicly available in the Gene Expression Analysis Services (GEAS) Repository at http://dcm.ffclrp.usp.br/lssb/geas. Additionally, we have used a number of the developed services to create different integrated analysis scenarios to reproduce parts of two gene expression studies documented in the literature. The first study involves the analysis of one-color microarray data obtained from multiple sclerosis patients and healthy donors. The second study comprises the analysis of RNA-Seq data obtained from melanoma cells to investigate the role of the remodeller BRG1 in the proliferation and morphology of these cells. Our methodology provides concrete guidelines and technical details in order to facilitate the systematic development of semantic web services. Moreover, it encourages the development and reuse of these services for the creation of semantically integrated solutions for gene expression analysis.

## Introduction

Gene expression analysis has become a powerful tool for acquiring new knowledge about the mechanisms involved in a number of biological processes and diseases. Gene expression studies usually require the execution of various analysis activities on data, including normalization, identification of differentially expressed genes, cluster analysis and functional analysis, among others. Different software tools have been developed to support one or more of these activities individually.

Gene expression analysis usually demands the integrated use of a number of analysis tools. The integration of a series of tools to carry out an analysis process requires users to manually transfer data between different tools and/or to convert data formats due to data structural differences. This process can be time consuming and error prone due to the increasing number of analysis tools and data formats available in this domain [1, 2]. Therefore, the availability of integrated environments is highly desirable in this domain.

A number of research platforms have been proposed for the (semi) automatic integration of computational resources in the functional genomics domain [3–7]. These genomic research platforms enable the (semi) automatic interconnection and execution of a number of activities defined as part of an analysis process. In general, they assist users in the execution of an analysis process by individually performing each process activity and automatically transferring data between different tools in order to exempt users from the underlying details of the integration process [1, 8]. Genomic research platforms can benefit from the explicit use of semantics to improve their integration capabilities. For example, the creation of workflows in Galaxy [7] could be improved by augmenting tool descriptions with semantic annotations, thus yielding to the creation of semantically integrated workflows.

Some genomic research platforms can not be easily extended in order to cope with new tools and data types [1]. In order to overcome this limitation, an increasing number of analysis tools in this domain have been developed as, or adapted to, web services [9–13]. Web services provide standardized programming interfaces, which facilitate programmatic access to tools and interoperability between different computer resources, therefore facilitating data/tool integration.

The integrated use of a set of web services to perform a more complex task is called service composition. Currently, one of the main goals of the web services research community is to enable automated support for service composition. In order to facilitate the development of web services and their (automated) composition, data produced and/or consumed by these services are usually specified using a standard machine-parsable language, such as HTML, XML or JSON. The use of such languages facilitates the automatic interconnection of services sharing syntactically compatible data. Nevertheless, different languages can be used to specify data produced and/or consumed by a web service. Further, the same data can even be represented differently using the same language. In this context, automatic composition of services with syntactically incompatible data is unfeasible, regardless of actual data meaning. Thus, automated service composition requires explicit definition of semantics to enable accurate interconnection of services and data exchange [14, 15]. Once there is an agreement on the meaning of exchanged data and service operations, data can easily be transformed from one representation to another thus creating semantically integrated service compositions.

The Semantic Web consists in a web of structured data that can be processed and comprehended by machines [16]. Semantic web technologies enable the assignment of semantics to structured data published on the Web through the use of ontologies, which are computational resources used to represent knowledge in specific domains [16, 17]. Ontologies provide a set of concepts and associated relationships explicitly defined in some formal logic. Semantic web services consist of web services to which semantics has been assigned by using ontologies [18]. The semantic enrichment of services enables the assignment of unambiguous meaning to data and service operations.

In general, semantic web services can be accessed through message exchange based on standards and architectural styles, such as Simple Object Access Protocol (SOAP) [19] and Representational State Transfer (REST) [20], respectively. Different approaches have been proposed for semantically describing both SOAP-based and RESTful web services, such as OWL-S [21], SAWSDL [22] and SA-REST [23].

In recent years, a number of approaches have been defined for the development of web services from legacy software tools, and for the semantic annotation of web services. Most of these approaches can be considered model-driven, which are usually domain-specific. However, to the best of our knowledge, no suitable approach has been defined for the functional genomics domain. In addition, these approaches lack concrete guidelines for the creation of web services from existing analysis tools, in general, and for the creation of RESTful web services, in particular. Furthermore, they also lack support for the semantic annotation of web services.

In this paper, we present a methodology for the development of RESTful semantic web services for gene expression analysis. This methodology aims at providing a systematic approach for both the implementation of RESTful web services from existing analysis tools and the semantic annotation of these services. In contrast to other existing approaches [24, 25], our methodology relies on state-of-the-art standard technologies and provides a fair amount of technical details thus facilitating its application. In order to illustrate its usage, we have applied the proposed methodology to the development of a number of RESTful semantic web services for gene expression analysis. Finally, we have integrated some of the developed services to create two frequently used gene expression analysis scenarios. The first scenario was used to reproduce part of a study already documented in the literature involving the analysis of one-color microarray data obtained from multiple sclerosis patients and healthy donors [26]. The second scenario was also used to reproduce part of a study involving the analysis of RNA-Seq data to investigate the role of the remodeller transcription activator BRG1 gene in the proliferation and morphology of melanoma cells [27]. The proposed scenarios emphasize the importance of our methodology to encourage the development and reuse of services towards the creation of integrated solutions for gene expression analysis.

## Methods

We carried out the following steps in order to define our methodology: 1) study of technologies for the development of RESTful semantic web services; 2) study of gene expression technologies and analysis tools; 3) definition of activities and concrete guidelines for the development of RESTful semantic web services from existing software tools; 4) application of the proposed methodology to the development of a number of RESTful semantic web services for gene expression analysis; 5) definition of two integration scenarios including some previously developed web services for the integrated analysis of gene expression data.

### RESTful web services

Representational State Transfer (REST) [20] is a client-server architectural style that provides a behavioral model for client applications and web services. REST describes a number of design principles and constraints, such as stateless communication and the use of uniform interfaces and self-descriptive messages that should be applied in REST-based services.

Services developed according to the REST style are usually referred to as RESTful web services. Each RESTful web service should expose a set of resources, i.e., any type of information exclusively identified by a Uniform Resource Identifier (URI) that can be referenced through the web. The access to a resource occurs through a uniform interface that provides standard access methods for handling the resource. When a particular resource is accessed by a client application, a representation of this resource reflecting its current state should be returned in response to the request. The decoupling of a resource from its representation enables a service provider to return different representations for the same resource in order to provide enhanced flexibility for different client applications. In general, resources are represented in either Extensible Markup Language (XML) or JavaScript Object Notation (JSON) formats.

RESTful web services provide uniform interfaces accessed only through HTTP methods, so that interoperability among different web services can be accomplished, and the development of client applications to interact with RESTful web services is simplified. Moreover, REST-based technologies provide a simple, lightweight interaction model and have been increasingly used in the development of web services [28, 29].

### Semantic Annotations for WSDL (SAWSDL)

Web Services Description Language Version 2 (WSDL 2.0) [30] is an XML-based language that can be used for the description of web services, including RESTful services. The WSDL description of a RESTful web service specifies the operations provided by the service, the formats of input and output messages, the technical details to communicate with the service and the service location. Since WSDL is a machine-readable language, it enables client applications to interpret service descriptions and automatically interact with services.

Although WSDL enables the syntactical description of different service features, it does not provide any intrinsic mechanism for the association of semantics to WSDL components. To cope with this limitation, the Semantic Annotations for WSDL (SAWSDL) [22] approach was created to provide mechanisms for the semantic annotation of WSDL components. This approach defines a set of extension attributes that can be used to semantically annotate WSDL service descriptions. Basically, three extension attributes are defined in this approach: *modelReference*, *liftingSchemaMapping* and *loweringSchemaMapping*.

The *modelReference* extension attribute can be used to associate a WSDL or XML Schema element to one or more ontological concepts. This attribute can be used to annotate XML Schema type definitions, element declarations and attributes, as well as WSDL interfaces, operations and faults. The annotations are used to provide a description of the main components of WSDL or XML Schema documents based on a set of concepts formally defined in an ontology. Thus, the *modelReference* attribute is useful for semantically describing input/output data, as well as the functionality provided by a service.

The *liftingSchemaMapping* and *loweringSchemaMapping* extension attributes can be used to associate XML Schema components to lifting and lowering mappings, respectively. A lifting mapping enables the transformation of XML structures contained in service messages into ontological concepts, whereas a lowering mapping enables the transformation of ontological concepts into concrete XML messages. These mappings enable different XML structures from client applications and services to be mapped onto ontological concepts in order to resolve syntactical differences, allowing the communication between client applications and services.

### Gene expression analysis

In order to apply the proposed methodology we have selected different software resources to be wrapped by RESTful semantic web services for the analysis of gene expression data. The following resources were considered: affy (version 1.44.0) [31] and limma (version 3.22.3) [32] R packages, used for one/two-color microarray data preprocessing using Robust Multi-array Average (RMA), subtract, loess and average quantile methods; R standard libraries (version 3.1.1), used for the identification of differentially expressed genes in one-color and two-color microarray data using fold change or Student’s t test analysis; the DESeq2 R package (version 1.6.3) [33], used for the identification of differentially expressed genes in RNA-Seq data using a model based on the negative binomial distribution; Cluster 3.0 [34], used for hierarchical and k-means clustering of microarray data (one-color or two-color); Java TreeView 1.1.6 [35], used for visualization of hierarchically clustered microarray data; the gProfileR R package (version 0.5) [36], used for enrichment analysis of gene expression data using the hypergeometric statistical test; the SOAP-based DAVID web service (version 1.1) [13], used for enrichment analysis of gene expression data using the Fisher’s Exact test; the gage R package (version 2.16.0) [37], used for gene set enrichment analysis of gene expression data; and the pathview R package (version 1.6.0) [38], used for visualization of gene expression data rendered into KEGG pathways [39].

We have then defined two scenarios for integrated analysis of gene expression data using some of the developed services. Each scenario was implemented as a web application. These web applications were developed using Javascript [40], jQuery (https://api.jquery.com/) and Java Servlet [41] technologies. In the first scenario, one-color microarray data were initially normalized and then a set of differentially expressed genes was identified. In the sequel, relevant biological pathways/processes related to the differentially expressed genes were identified and, finally, relevant KEGG pathways were visually analyzed. Agilent Whole Human Genome one-color microarray data, available in ArrayExpress under the accession number E-MEXP-3905, was obtained from Mesenchymal Stromal Cells (MSCs) that were isolated from a group of patients diagnosed with Multiple Sclerosis (MS) and treated with high dose immunossupression followed by Autologous Hematopoietic Stem Cell Transplantation (AHSCT) [26]. Pre-transplantation MSCs samples, post-transplantation MSCs samples, as well as healthy controls MSCs samples were used in this study.

In the second scenario, a set of differentially expressed genes was initially identified from RNA-Seq data. The relevant biological processes related to the differentially expressed genes were then identified and analyzed. Illumina HiSeq 2500 based RNA-Seq data, available in Gene Expression Omnibus (GEO) under accession number GSE61966, was obtained from 501Mel melanoma cells to address the function of the remodeller BRG1 in the proliferation and morphology of these cells [27]. In order to investigate the role of BRG1, the authors performed the si/shRNA knockdown of both BRG1 and MITF genes and performed a comparative analysis. BRG1 knockdown cell samples, MITF knockdown cell samples, as well as control samples were used in this study.

## Results

### Methodology for the Development of RESTful SWS

This section presents our methodology for the systematic development of RESTful semantic web services from existing software tools. This methodology consists of four general activities: (i) create a RESTful web service from an existing software tool; (ii) automatically generate a WSDL service description from the service implementation; (iii) define domain and service ontologies; and (iv) use these ontologies to semantically annotate the WSDL service description according to the SAWSDL approach. Each of these activities is discussed in the sequel and the application of the methodology is illustrated in section *Methodology Application*.

#### Service Implementation

This activity aims at implementing a RESTful web service as a wrapper of a software tool, i.e., software application, service or library component, in order to expose a set of existing functions as a web service without modifying the implementation of the software tool.

In software engineering, a design pattern is a generic solution that can be applied to solve a particular type of recurring problem in the development of different software systems. A design pattern provides an abstract description of a set of components and their interactions that can be used to guide the development of a particular software system. The creation of wrappers can be carried out according to the well-defined Adapter design pattern [42].

**Fig 1:**
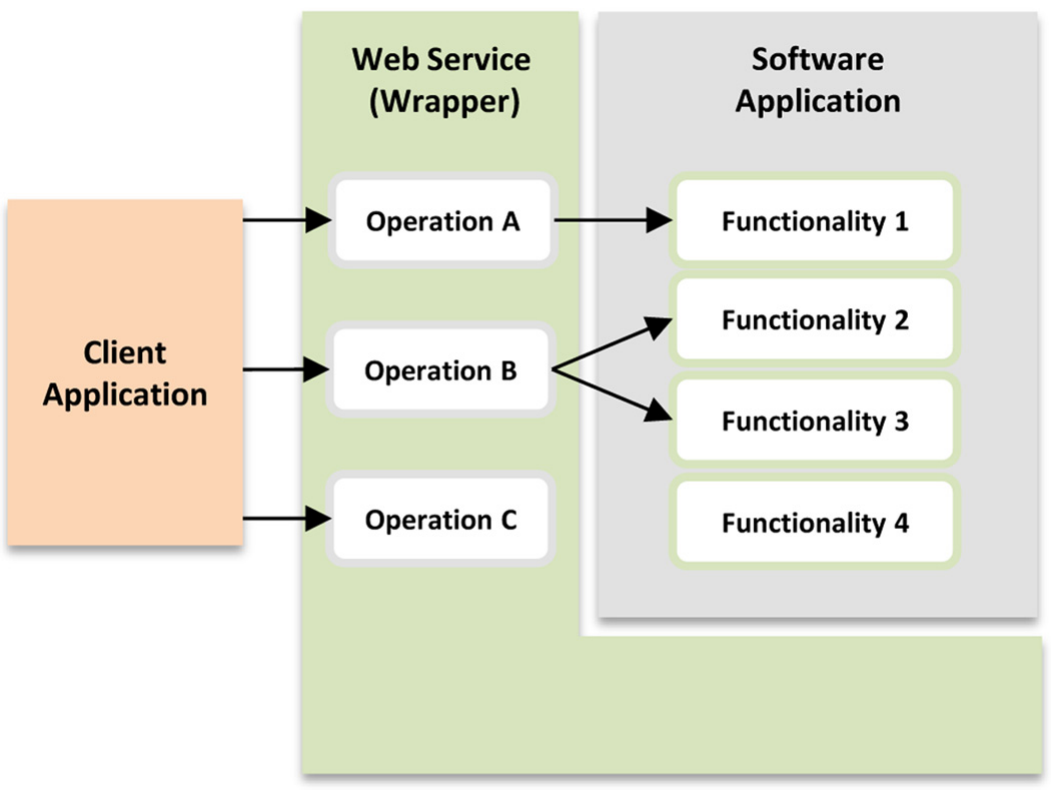
Architecture of a generic web service (wrapper). Service operations A and B provide access to different functions of an existing software application, while the service operation C was independently implemented of the tool being wrapped. Functionality 4 of the software application is not accessed by the service.

When wrapping a software tool into a web service, a subset of the functions provided by this tool can be accessed by one or more service operations. The service designer may disregard some functions of the tool when designing the wrapper, due to reasons possibly related to the way in which the wrapper is expected to be used. Moreover, additional operations, which do not directly access the tool itself, can also be defined by the service developer in order to enable proper remote access to the tool. For example, a service developer may have to define an operation to manage the execution of an analysis been performed by the tool or to return the result of a completed analysis. Fig 1 shows the architecture of a generic web service implemented as a wrapper of an existing software application.

The implementation of a wrapper service can be carried out according to the following steps:

*1. Selection of core functions*: the existing software tool can provide a number of different functions. This step aims at identifying and selecting a subset of these functions to be accessed through the wrapper service. In this step, we create a functional specification listing the main functions of the existing tool. This list provides a better understanding of the functions provided by the tool and also serves as a basis for selecting the functions to be accessed by the service.
*2. Definition of interaction model*: this step aims at defining an interaction model to describe the set of interactions between the service under development and the software tool being wrapped, and between the service and potential client applications. The development of an interaction model can be carried out in two steps through the creation of UML use cases and sequence diagrams [43, 44].

In the first step, we create a set of use cases in order to capture in an abstract way information regarding the execution of the relevant functions to be provided by the service under development. Each use case describes the required interactions between external entities (client application and software tool being wrapped) and the service to achieve a defined goal. These interactions are described through the specification of a typical course of events and, possibly, alternative courses of events. During the definition of the use cases, each identified core function of the existing software tool is associated with at least one use case.

In the second step, we create a set of UML sequence diagrams from the previously created use cases in order to capture the set of concrete interactions that should occur between the external entities and the service being developed. The creation of sequence diagrams aims at identifying the set of operations that should be provided by the service. During this step, we create at least one sequence diagram for each typical course of events defined in each use case. Each event defined in the typical course of events is mapped to a message in the sequence diagram, so that the temporal order of message exchange in the sequence diagram follows the course of events defined in the use case. If alternative courses of events have been defined in a use case, a sequence diagram can also be created to represent each one of them.

During the definition of the interaction model, each identified core function of the existing software tool is mapped to at least one service operation. There are four basic types of RESTful web service operations, generally referred as CRUD operations: CREATE, used for the creation of a resource on the server; READ, used for the retrieval of a resource stored on the server; UPDATE, used for the update of a previously stored resource on the server; and DELETE, used for the removal of a previously stored resource on the server. These different types of service operations can be used separately or can be combined in the definition of an interaction model.

Additionally, the stateless nature of RESTful web services should be taken into account during the definition of the interaction model. According to this characteristic, request messages sent from a client application to a service provider must be self-descriptive, i.e., they must contain all the information needed for the provider to handle the request. As a consequence, some mechanism should be used to simulate client sessions if necessary. One example of such a mechanism is the use of an identifier to uniquely associate a virtual client session to a workspace (server directory) so that each workspace is accessible only through its unique identifier. Each workspace then maintains all information related to a unique client section, including submitted and processed data, as well as the task execution status.

*3. Implementation of service operations*: once the interaction model has been defined, we implement all service operations defined in the model. In the context of this work, the implementation of the service operations has been carried out using the Java API for RESTful Web Services (JAX-RS) [45].

A RESTful web service is implemented as a Java class, and each service operation is implemented as a separate method of this class, responsible for performing the required service. Each method implementation embodies any necessary calls to the application core functions, corresponding to one or more methods of the application API. Communication between the RESTful service and the software resource being wrapped can be implemented using the Java Native Interface (JNI) programming interface (http://docs.oracle.com/javase/7/docs/technotes/guides/jni/), which enables Java applications to communicate with applications and libraries implemented in other programming languages.

Once the service operations have been implemented, we need to explicitly map each operation to a corresponding HTTP method. Particularly, the CREATE, READ, UPDATE and DELETE operations should be mapped to the POST, GET, PUT and DELETE methods of the HTTP protocol, respectively. This mapping can be carried out using the @POST, @GET, @PUT and @DELETE annotations provided by the JAX-RS API, respectively.

According to the REST model, any interaction with a RESTful web service is intended to access a resource. Thus, each service should provide a set of web resources exclusively identified by URIs. The @Path annotation provided by the JAX-RS allows the assignment of URIs to resources. The JAX-RS API provides a number of additional annotations that can optionally be associated to the implemented Java methods. For example, the @PathParam annotation is used to extract values from parameters embedded in URIs and convert them to parameters of the associated Java method.

*4. Test of the developed RESTful web service*: This step aims at verifying whether the service behavior has been properly implemented and correcting possible implementation errors. In this step, we implement a (simple) client application to interact with the developed service only for test purposes. Simple client applications can be automatically created using the Jersey Reference Implementation of JAX-RS (http://jersey.java.net/).

During this step, all service operations are individually tested in order to ensure that the behavior of each operation has been properly implemented and the expected results are produced. Since the RESTful service is implemented as a wrapper for an existing software application, we also ensure that the communication between a service operation and the associated functionality provided by the existing application has been properly implemented. Finally, after testing each individual service operation, we test each execution sequence identified during the definition of the interaction model in order to ensure that the overall service behavior has been properly implemented.

#### Service Description Generation

This activity aims at automatically generating a WSDL 2.0 service description from the Java service implementation. In order to perform this activity, the Java2WSDL tool provided as part of the Apache Axis2/Java project (http://axis.apache.org/axis2/java/core/) can be used. This tool can be accessed through the Axis2 Code Generator plug-in for Eclipse. Optionally, the functionality provided by Java2WSDL can also be accessed through different tasks defined in an Ant script, so that automation can be improved.

Since WSDL 2.0 can be used for the description of SOAP-based and RESTful web services, the generated service description contains three bindings, namely SOAP1.1, SOAP1.2 and HTTP. Since the SOAP bindings are not used for the developed RESTful service, they can be ignored. These SOAP bindings and their references can be removed if the designer wants to clean up the generated WSDL document, either manually, or by using a Java parser for the manipulation of WSDL 2.0 documents provided by the Apache Woden project (http://ws.apache.org/woden/). This parser can be also used to validate the generated WSDL 2.0 service description.

**Fig 2:**
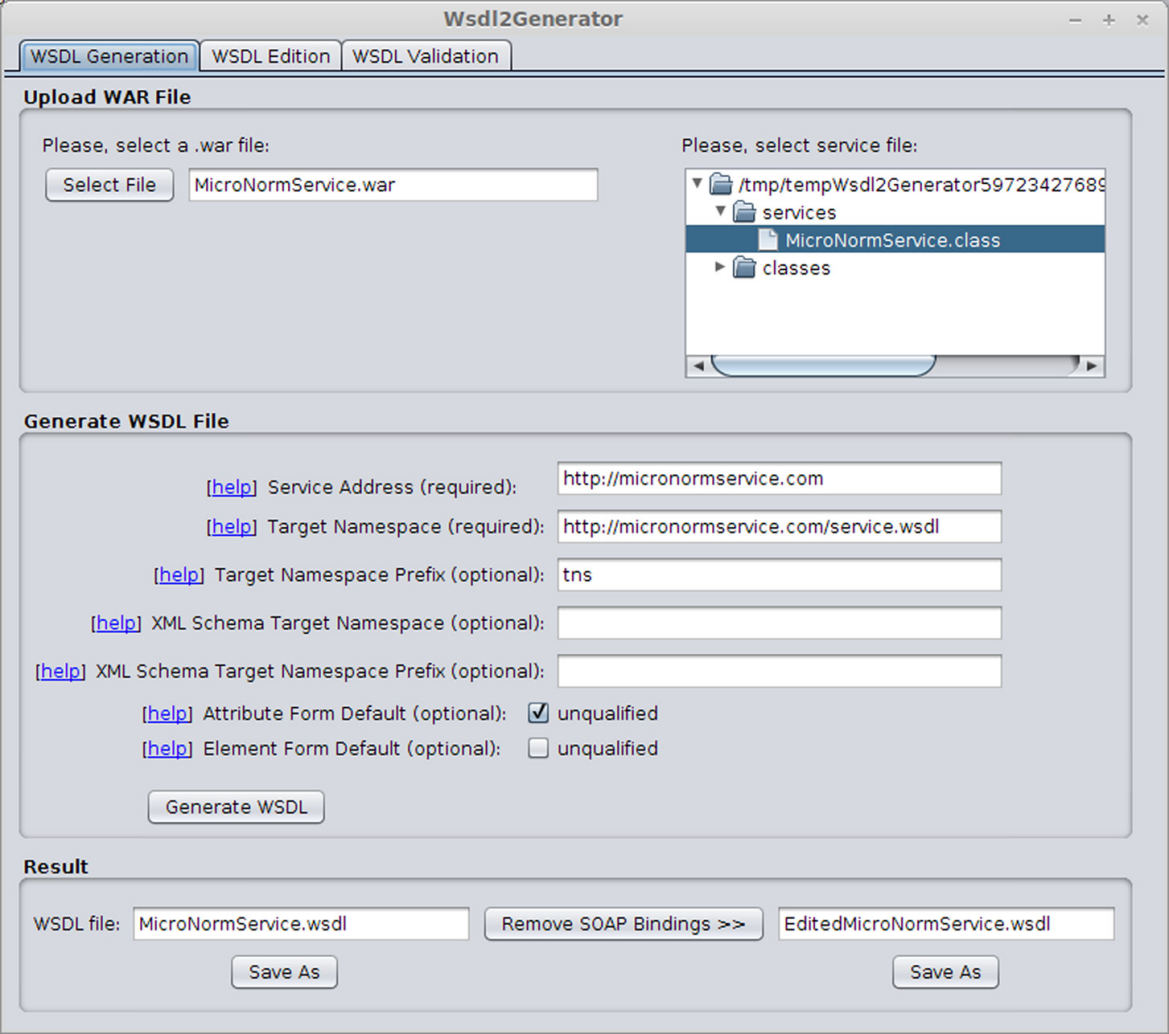
Screenshot of the Wsdl2Generator support tool.

In order to facilitate this activity, we have also developed a Java support tool, named Wsdl2Generator, for the creation, edition and validation of WSDL 2.0 service descriptions. Fig 2 displays a screenshot of our Wsdl2Generator tool.

#### Ontology Definition

This activity aims at defining domain and service ontologies, and creating cross-references between them. First, a domain ontology is defined to provide concepts and associated relationships that describe the knowledge domain to which the service under development belongs. Since domain ontologies describe well-established knowledge domains, they are usually stable and can be used in the development of a number of services pertaining to the domain. During this activity, we initially search for a suitable ontology. However, in case no suitable domain ontology is identified, an appropriate domain ontology is developed, preferably by reusing existing ontologies. Ontology development is carried out with the assistance of a domain specialist.

Second, a service ontology is defined to provide concepts and associated relationships that describe the core functions provided by the service under development, including concepts related to required inputs and expected outputs. Service ontologies, in contrast, are not as stable as domain ontologies and they often need to be extended to cope with new services pertaining to the same knowledge domain.

Finally, after the definition of the domain and service ontologies, we define cross-references between these two ontologies. In general, the definition of cross-references between concepts from different ontologies allows the qualification or semantic enhancement of the concepts. The definition of cross-references aims at semantically enhancing the concepts provided by the service ontology with concepts from the domain ontology. This activity is required because some concepts defined in the service ontology may have (slightly) different meanings across various knowledge domains.

Different methodologies have already been proposed for the development of ontologies. A comprehensive review of the existing methodologies for ontology development can be found at [46, 47]. The development process used for the creation of the domain and service ontologies is out of the scope of this work. In this work, we specify the domain and service ontologies using the Web Ontology Language Version 2 (OWL 2) [48]. An ontology written in OWL can be serialized and shared using a number of distinct syntaxes, including the RDF/XML syntax. The development of such ontologies can be carried out using the Protégé [49] ontology editor, which enables the design, editing and visualization of ontologies.

#### Semantic Annotation of Service Description

This activity aims at semantically annotating the generated WSDL 2.0 service description according to the SAWSDL approach. The semantic annotation of a service description is also carried out with the support of a domain specialist, who has the necessary knowledge to accurately map semantic information to the service description.

The semantic annotation of service descriptions according to the SAWSDL approach can be carried out using three extension attributes, namely *modelReference*, *liftingSchemaMapping* and *loweringSchemaMapping*. The semantic annotation of WSDL service descriptions using SAWSDL can be performed manually. However, the use of a supporting tool, such as Radiant [50], facilitates this activity. Optionally, the semantic annotations can be specified using graphical languages, such as UML, to facilitate the visualization of the annotations.

In our methodology, we prescribe the application of the *modelReference* attribute to WSDL operations defined in the WSDL interface component in order to associate ontological concepts that describe the functions provided by the service with these WSDL elements. General-purpose service operations, such as operations for the submission, retrieval or removal of data, do not need to be annotated, in contrast with their inputs and outputs, which are annotated.

The *modelReference* attribute is also applied to simple/complex XML Schema type definitions defined in the WSDL types component in order to associate ontological concepts describing the service required inputs and expected outputs with these type definitions. When a top-level XML Schema element is declared in terms of simple types, we consider that only the simple types themselves need to be annotated with the *modelReference* attribute. This approach takes into account that a simple type annotation also applies to any top-level element using that type definition.

When a top-level XML Schema element is declared in terms of complex types, the annotation of its complex types can be carried out in two alternative ways: at the member element level and at the complex type container level. In the former, all member elements contained in a complex type are annotated with the *modelReference* attribute, while in the latter, the complex types themselves are annotated. Furthermore, it is possible to combine these two approaches since these types of annotations are independent. In this case, the complex types themselves and also their member elements are annotated with the *modelReference* attribute.

**Fig 3:**
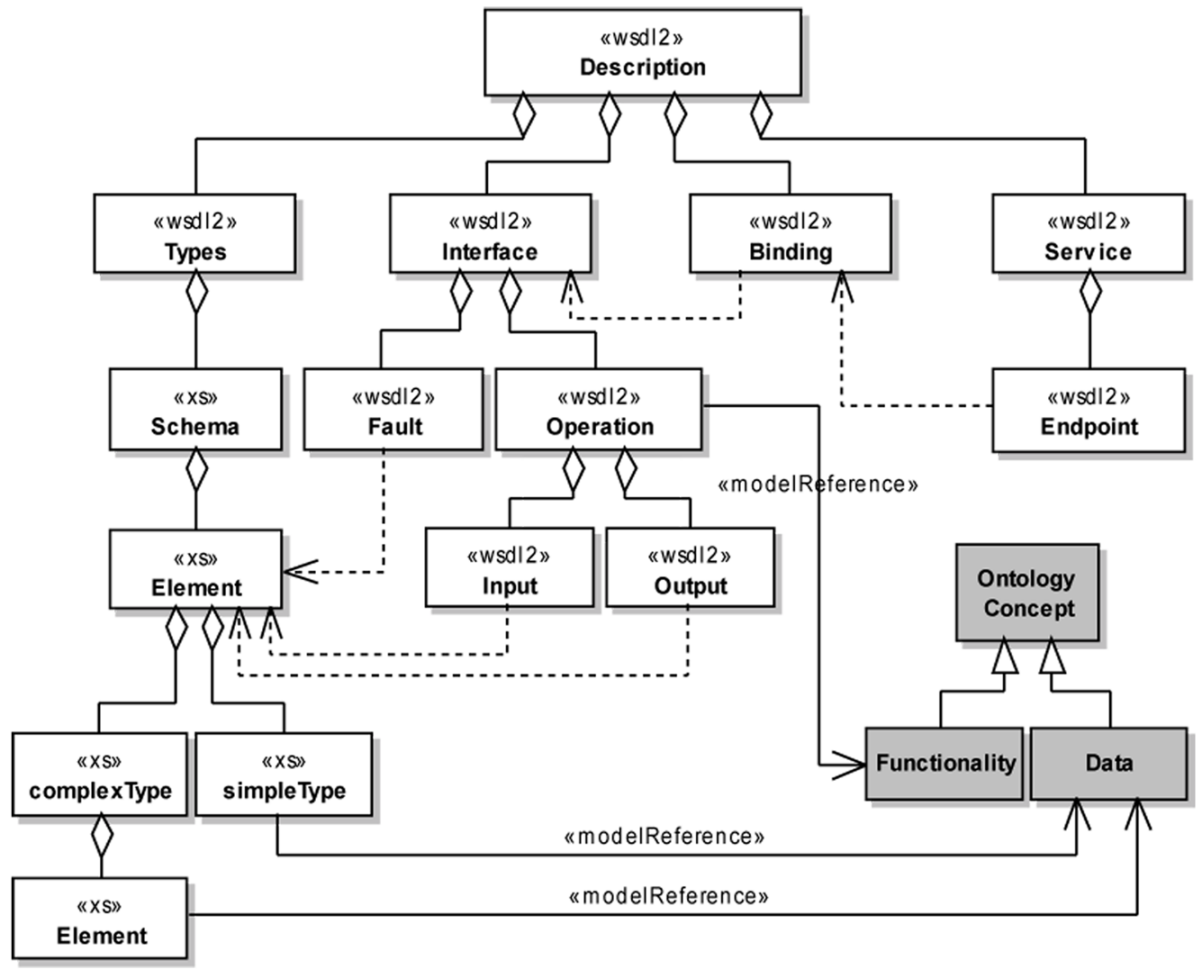
Annotation of WSDL elements using the *modelReference* attribute according to the proposed methodology. A named rectangle represents a UML class. A white class represents a WSDL element, while a gray class represents a service ontology element. A directed dashed line (UML dependency) indicates that a WSDL element references another one. A solid line with an hollow diamond (UML aggregation) is used to indicate a WSDL element contained as part of another one. A solid line with an hollow triangle as an arrowhead (UML generalization) represents a subsumption relation between ontology concepts. Finally, a directed solid line (UML association) stereotyped as < < modelReference > > represents the use of the *modelReference* attribute to annotate a WSDL element with an ontology concept. Each general-purpose WSDL operation is associated to an ontological concept representing a service functionality. Each simple/complex XML Schema type definition is associated to an ontological concept representing a data format. The annotation of complex types is presented only at the member element level.

Fig 3 presents a UML class diagram representing the main components of a WSDL service description and the annotation of these components with the *modelReference* attribute. These components are annotated with the *modelReference* extension attribute to associate them with concepts from the service ontology. Although this extension attribute can be applied both to XML Schema type definitions, element declarations and attributes, and to WSDL interfaces, operations and faults, we consider the annotation of XML Schema top-level element declarations and attributes, as well as of WSDL interfaces and faults, as an optional step in the context of this work.

Optionally, the *liftingSchemaMapping* and *loweringSchemaMapping* extension attributes can be used to associate XML Schema components to lifting and lowering schema mappings, respectively. Usually, lifting schema mappings can be defined in XSLT [51] and lowering schema mappings can be defined using SPARQL [52] and XSLT. *SchemaMapping* attributes can be applied to XML Schema type definitions and/or element declarations in order to enable semantic data exchange between a potential client application and the service provider. The annotation of a XML type definition with a *SchemaMapping* attribute is only propagated to the top-level element of that type if the element itself does not declare any schema mapping. This mechanism allows types to provide generic schema mappings, and elements to specify more concrete mappings appropriate for the specific use of that type definition.

After the semantic annotation of the WSDL 2.0 service description, the parser provided by the Apache Woden project or the Wsdl2Generator tool can be used again to validate the annotated service description.

### Methodology Application

After the definition of our methodology, we have applied this methodology to the development of a number of RESTful semantic web services for gene expression analysis. This section discusses in details the application of the proposed methodology in the development of a RESTful semantic web service, called KeggPathwayViewer, for gene expression data rendering into KEGG pathway graphs.

#### Service Implementation

The KeggPathwayViewer service wraps the pathview R package [38]. This package provides a number of functions, but we have selected only one of them to be accessed via the wrapper service, namely the function that enables mapping and rendering of gene expression data into KEGG pathway graphs.

**Fig 4:**
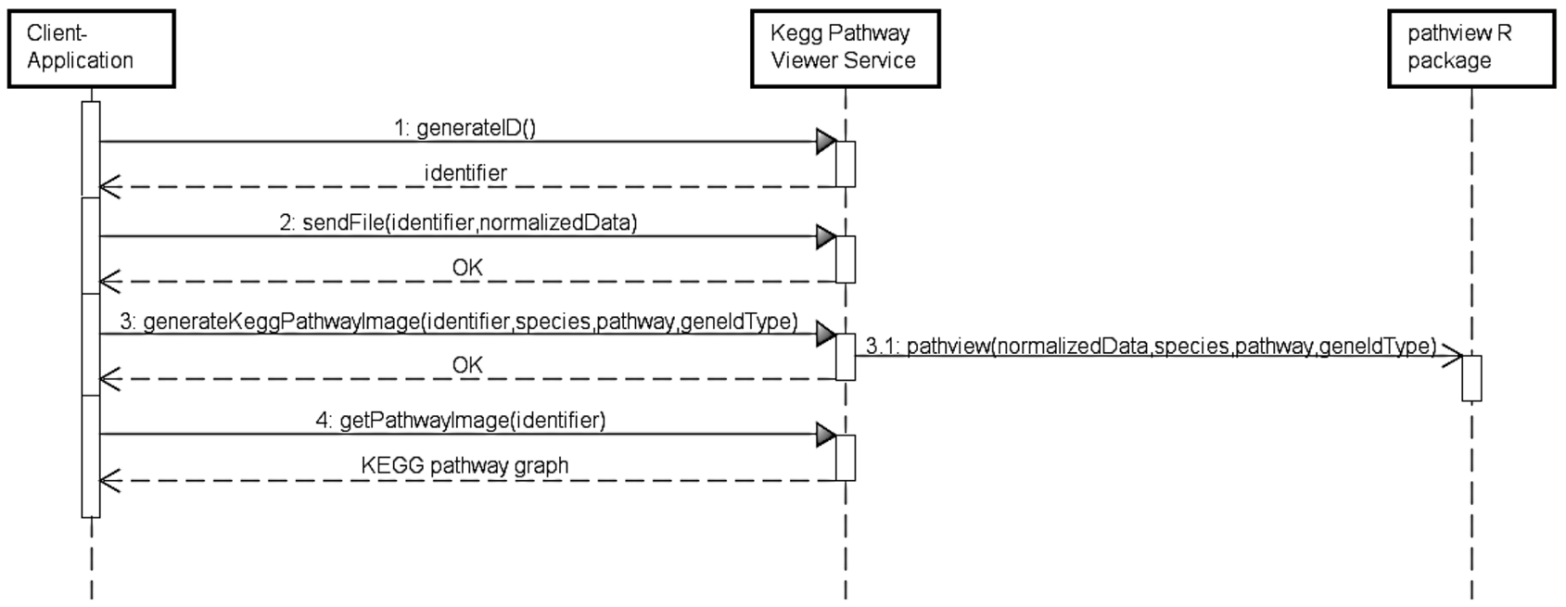
KeggPathwayViewer service interaction model. Each named rectangle represents an individual participant in an interaction. A (dashed) vertical line following the rectangle represents the lifetime of the participant. A line connecting the lifetimes of two participants represents a message exchanged between participants. A solid line with a solid arrowhead at its end represents a synchronous communication (call and reply). A solid line with a stick arrowhead at its end represents an asynchronous communication.

During the definition of an interaction model for the KeggPathwayViewer service, a single UML use case was initially defined containing a typical course of events and two alternative courses of events. Then, a UML sequence diagram was defined for each course of events previously defined in the use case. Fig 4 presents the UML sequence diagram defined for the typical course of events associated with this service. This diagram presents the ordered sequence of interactions that should occur between the external entities (client-application and pathview R package) and the KeggPathwayViewer service for rendering gene expression data into a KEGG pathway graph.

The developed interaction model contains eight operations: *generateID*, *sendFile*, *generateKeggPathwayImage*, *getSpecies*, *getPathways*, *getGeneIdentifierTypes*, *getStatus* and *getPathwayImage*. All operations were then implemented using the JAX-RS API. Most operations were implemented as *READ* operations, since they allow the retrieval of different resources stored on the server. Exceptions are operations *sendFile* and *generateKeggPathwayImage*, which were implemented as *CREATE* operations, since they allow the creation of different resources on the server. Operation *generateKeggPathwayImage* was directly mapped to the identified core functionality of the pathview R package.

Operation *generateID* creates a workspace (server directory) associated with a unique identifier used to store normalized gene expression data to be analyzed by the service and the results generated by the analysis, and returns this identifier.

Operation *sendFile* enables the submission of a text file containing normalized gene expression data. This operation requires as input the unique identifier provided by operation *generateID*, a text file to be stored (File object) and a filename.

Operation *generateKeggPathwayImage* maps and renders previously submitted normalized gene expression data into a KEGG pathway graph. This operation requires as input the identifier provided by the operation *generateID*, an identifier representing a biological species, an identifier representing a KEGG pathway and a gene identifier type. As a result of this operation, a PNG image that represents gene expression data into a KEGG pathway graph is generated. The image itself is produced by an external tool named R Server using the pathview package and then stored in the service workspace. Communication between Java and R is carried out using the Rserve package [53] (http://cran.r-project.org/web/packages/Rserve/index.html), which allows the integration of programs written in Java with the R Server using a TCP connection.

The implementation of resource representations in XML was facilitated using the Java Architecture for XML Binding (JAXB) [54] API, which provides mechanisms for handling XML documents in Java. Operations *getSpecies*, *getPathways* and *getGeneIdentifierTypes* return JAXB objects that represent XML files. The JAXB object returned by operation *getSpecies* contains a list of identifiers representing all biological species that can be analyzed. The JAXB object returned by operation *getPathways* contains a list of identifiers representing all KEGG pathways that can be generated for a particular species. Finally, the JAXB object returned by operation *getGeneIdentifierTypes* contains a list of gene identifier types that can be specified in normalized gene expression data for a particular species. Operations *getPathways* and *getGeneIdentifierTypes* require as input an identifier representing a biological species.

Operation *getStatus* requires as input the identifier provided by operation *generateID*, and returns the execution status of an analysis being performed by the service.

Finally, operation *getPathwayImage* allows the retrieval of the resulting PNG image that represents gene expression data into a KEGG pathway graph. This operation should be invoked only when the analysis is successfully finished. Operation *getPathwayImage* requires as input only the identifier provided by the operation *generateID*.

The KeggPathwayViewer service should be used as follows. First, the operation *generateID* should be invoked to generate a unique identifier that should be provided as input for the subsequent operations. Next, the operation *sendFile* should be invoked. This operation is used to submit a text file containing normalized gene expression data. At this point, the operation *generateKeggPathwayImage* can be invoked to start the analysis of the submitted data. This operation requires tree parameters: an identifier representing a biological species, an identifier representing a KEGG pathway and a gene identifier type. In case the user needs help to set one or more of these parameters, the operations *getSpecies*, *getPathways* and *getGeneIdentifierTypes* could optionally be invoked before operation *generateKeggPathwayImage*. Once the operation *generateKeggPathwayImage* is invoked, the operation *getStatus* should be periodically invoked to verify the execution status of the analysis being performed. Finally, when the analysis is finished, i.e., the operation *getStatus* returns “finished”, the operation *getPathwayImage* should be invoked to retrieve the analysis result.

Once all service operations have been implemented as Java methods, they were mapped onto specific HTTP methods. Java methods *sendFile* and *generateKeggPathwayImage* were annotated with the @POST annotation provided by the JAX-RS API. All other Java methods were annotated with the @GET annotation. Moreover, all implemented Java methods were annotated with the @Path annotation. The @PathParam annotation was also associated to each parameter defined in the Java methods.

Lastly, a simple client application was implemented using the JAX-RS Jersey Reference Implementation to test the KeggPathwayViewer service.

#### Service Description Generation

After the service implementation, we automatically generated a WSDL 2.0 description for the service using the Java2WSDL tool, and we removed the SOAP bindings contained in the service description. Finally, we validated the generated WSDL 2.0 service description using the Wsdl2Generator tool.

#### Ontology Definition

In this step, we have developed two OWL ontologies: the Gene Expression Ontology (GEXPO) [55] and the Gene Expression Analysis Services Ontology (GEXPASO). The GEXPO ontology provides concepts and associated relationships to describe the functional genomics domain, which includes the description of the biological process of gene expression and related experimental processes for gene expression measurement, such as DNA microarrays [56] and RNA-Seq [57]. During the development of this ontology, most concepts that represent biomolecules involved in the described processes were reused from two other ontologies, namely the Gene Ontology (GO) [58] and the Sequence Ontology (SO) [59].

The GEXPASO ontology provides concepts and associated relationships to describe not only analysis processes commonly performed in gene expression data, but also structural and behavioral aspects of the service under development. The Software Ontology (SWO) (http://theswo.sourceforge.net/index.html) was developed to provide a framework for the description of software tools, so that we could use this ontology as the basis for the definition of the most general concepts of the GEXPASO ontology. Furthermore, after the definition of the GEXPASO ontology, we established a number of cross-references between this ontology and the GEXPO ontology.

**Fig 5:**
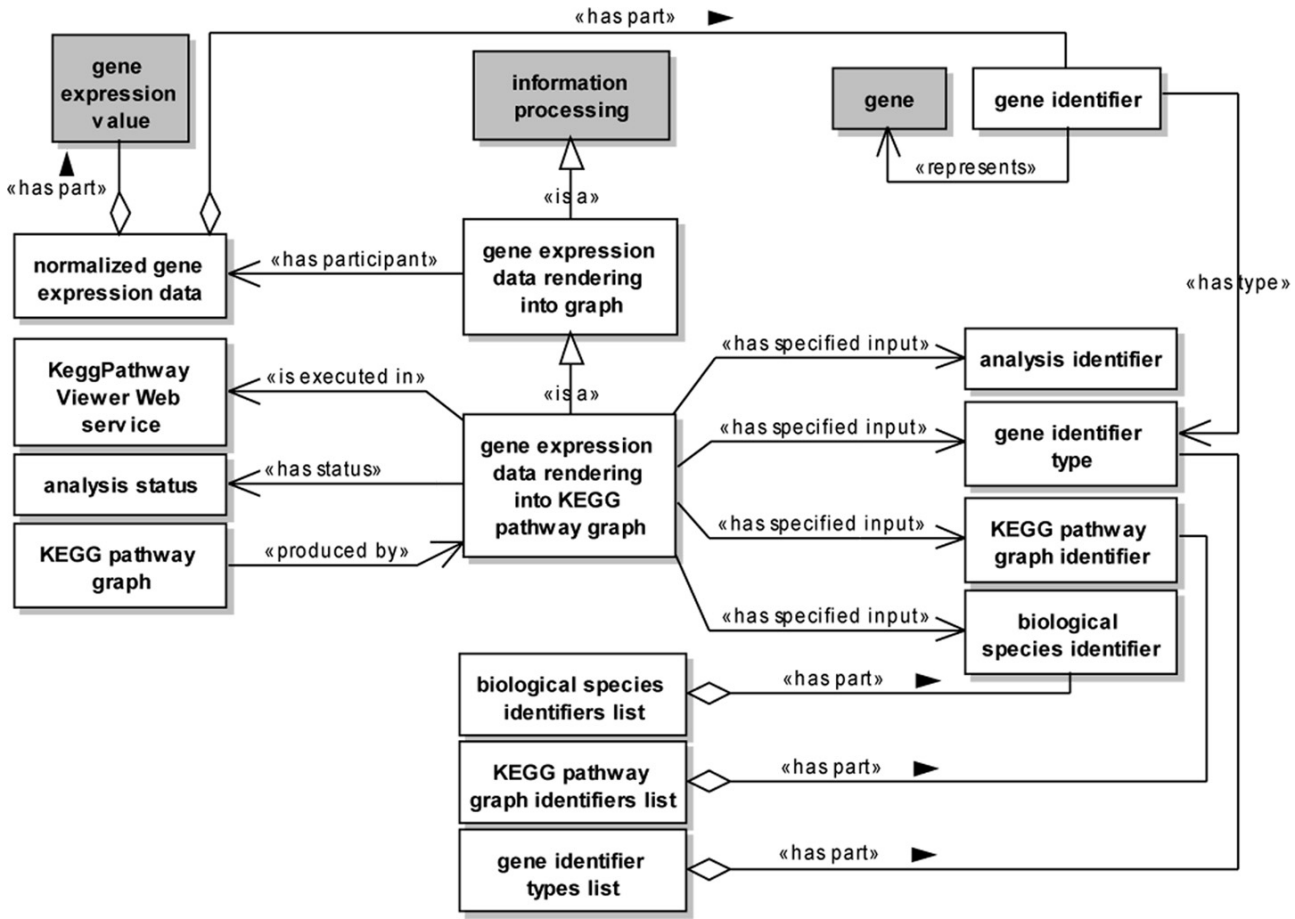
UML class diagram representing an excerpt of the GEXPASO ontology. A named rectangle represents a class. Classes in white represent concepts introduced in the GEXPASO ontology, while classes in gray represent concepts reused from other ontologies. A solid line with a stick arrowhead at its end represents a relationship between two concepts (UML association). A solid line with a hollow diamond at its end represents an aggregation relationship (UML aggregation). Finally, a solid line with an hollow triangle as an arrowhead represents a generalization relationship (UML generalization).

Fig 5 presents a UML class diagram representing an excerpt of the GEXPASO ontology that concerns gene expression data rendering into KEGG pathway graphs. Each concept of the ontology is represented by a UML class. Further, all relationships between concepts are represented using a notation similar to the one proposed in [60]. Fig 5 shows only the concepts and relationships that are relevant to the application of our methodology in the development of the KeggPathwayViewer service.

The class *gene expression data rendering into graph* (subclass of *information processing*) represents a process responsible for mapping and rendering gene expression data into a graph. This class is associated to the class *normalized gene expression data* through a *has participant* relation, indicating that the process of rendering gene expression data into a graph requires normalized gene expression data as participant. The class *normalized gene expression data* is associated to the classes *gene identifier* and *gene expression value* through *has part* relations, indicating that such data contain gene identifiers and expression values, respectively. The class *gene identifier* is associated to the class *gene* through a *represents* relation, indicating that gene identifiers are used to represent genes. Additionally, the class *gene identifier* is associated to the class *gene identifier type* through a *has type* relation, indicating that gene identifiers have associated types. Different types were modeled as instances of the class *gene identifier type*, including Entrez gene identifier, gene name and official gene symbol (not depicted in Fig 5).

The class *gene expression data rendering into graph* is specialized by the class *gene expression data rendering into KEGG pathway graph*, which represents the process of mapping and rendering gene expression data into a KEGG pathway graph. This class is associated to the classes *gene identifier type*, *analysis identifier*, *KEGG pathway graph identifier* and *biological species identifier* through *has specified input* relations, indicating that this process requires a gene identifier type and identifiers representing an analysis (workspace), a KEGG pathway graph and a biological species as input, respectively. Classes *gene identifier type*, *KEGG pathway graph identifier* and *biological species identifier* are associated to the classes *gene identifier types list*, *KEGG pathway graph identifiers list* and *biological species identifiers list* through *has part* relations, respectively. These relations indicate that these lists have one or more gene identifier types, KEGG pathway graph identifiers and biological species identifiers as their parts, respectively.

Additionally, the *has status* relation defined between classes *gene expression data rendering into KEGG pathway graph* and *analysis status* indicates that the process of rendering gene expression data into a KEGG pathway graph is associated to some status. Classes *gene expression data rendering into KEGG pathway graph* and *KEGG pathway graph* are associated through a *produced by* relation, indicating that a KEGG graph is produced by this type of process. Furthermore, the *is executed in* relation defined between classes *gene expression data rendering into KEGG pathway graph* and *KeggPathwayViewer Web service* indicates that the process of rendering gene expression data into a KEGG pathway is performed by the KeggPathwayViewer web service.

#### Semantic Annotation of Service Description

After the definition of the GEXPO and GEXPASO ontologies, we have semantically annotated the WSDL 2.0 service description using the Radiant plug-in for Eclipse. In order to demonstrate this step, we have used the *modelReference* extension attribute to associate WSDL operations, inputs and outputs to concepts from the GEXPASO ontology.

**Fig 6:**
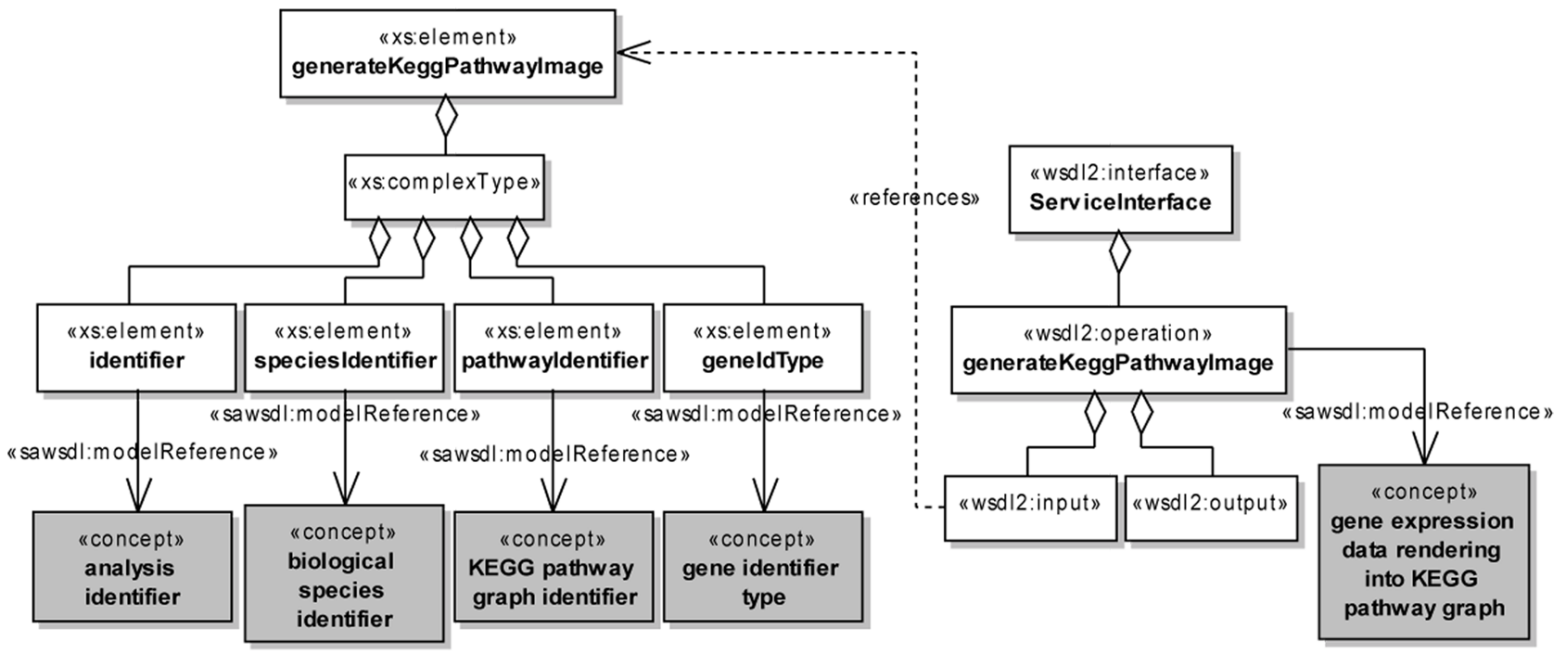
UML class diagram representing the semantic annotation of the WSDL operation generateKeggPathwayImage. A named rectangle represents a UML class. A white class represents a WSDL element, while a gray class represents a concept from the GEXPASO ontology. A directed solid line (UML association) stereotyped as < < *sawsdl:modelReference* > > represents the use of the *modelReference* extension attribute to annotate a WSDL element with a GEXPASO concept. A directed dashed line (UML dependency) stereotyped as < < *references* > > indicates that a WSDL element references another one. Finally, a solid line with an hollow diamond (UML aggregation) is used to indicate a WSDL element contained as part of another one.

Fig 6 shows a UML class diagram representing the syntactical structure and semantic annotation of WSDL operation *generateKeggPathwayImage*. This WSDL operation was associated to the GEXPASO concept *gene expression data rendering into KEGG pathway graph* and the input elements of this operation, identifier, speciesIdentifier, pathwayIdentifier and geneIdType, were associated to the GEXPASO concepts *analysis identifier*, *biological species identifier*, *KEGG pathway graph identifier* and *gene identifier type*, respectively.

After providing these semantic annotations, we validated the WSDL 2.0 service description once more using the Wsdl2Generator tool.

### Gene Expression Analysis Services (GEAS) Repository

We have developed a number of semantic web services to support different activities in gene expression analysis. The following services were developed to support the analysis of microarray data:

- *MicroNorm*, which allows the preprocessing of one- and two-color microarray data generated from three different platforms, viz., Affymetrix, Genepix (two-color) and Agilent (one-color). This service is implemented as a wrapper for the affy and limma R packages. The preprocessing of Affymetrix microarray data is carried out using the Robust Multi-array Average (RMA) method of the affy package. The preprocessing of Genepix (two-color) microarray data is carried out using the subtract, loess and Aquantile methods of the affy package. Finally, the preprocessing of Agilent (one-color) microarray data is carried out using the subtract, quantiles and avereps methods of the limma package;
- *MicroOneDifferentialAnalysis* and *MicroTwoDifferentialAnalysis*, which allow the identification of differentially expressed genes in one-color and two-color microarray data, respectively. Each of these services is implemented as a wrapper for different R standard libraries. The identification of differentially expressed genes is carried out in these libraries using fold change or Student’s t test analysis;
- *MicroHCluster*, which allows the hierarchical clustering of both one-color and two-color microarray data. This service is implemented as a wrapper for the Cluster 3.0 tool;
- *MicroKCluster*, which allows the k-means clustering of both one-color and two-color microarray data. This service is also implemented as a wrapper for the Cluster 3.0 tool;
- *MicroHClusterViewer*, which allows the visualization of hierarchically clustered microarray data. This service is implemented as a wrapper for the Java TreeView tool.

The following service was developed specifically to support the analysis of RNA-Seq data:

- *RnaSeqDifferentialAnalysis*, which allows the identification of differentially expressed genes in RNA-Seq data. This service is implemented as a wrapper for the DESeq2 R package. The identification of differentially expressed genes is carried out in this package using a model based on the negative binomial distribution.

Finally, the following services were developed to support the analysis of gene expression data obtained not only from microarray and RNA-Seq technologies but also other technologies:

- *EnrichmentAnalysis*, which allows the enrichment analysis of gene expression data. This service is implemented as a wrapper for the gProfileR R package. The enrichment analysis of gene expression data is carried out in this package using the hypergeometric statistical test;
- *DAVID-REST*, which also allows the enrichment analysis of gene expression data. This service is implemented as a wrapper for the SOAP-based DAVID web service. The enrichment analysis of gene expression data is carried out in the DAVID web service using the Fisher’s Exact statistical test;
- *GeneSetEnrichmentAnalysis*, which allows the gene set enrichment analysis of gene expression data. This service is implemented as a wrapper for the gage R package. The gene set enrichment analysis of gene expression data is carried out in this package using the method called Generally Applicable Gene-set Enrichment (GAGE);
- *KeggPathwayViewer*, which allows the mapping and rendering of gene expression data into KEGG pathways [39]. This service is implemented as a wrapper for the pathview R package.

We grouped these semantic web services into a publicly available service repository named Gene Expression Analysis Services (GEAS) Repository (available at http://dcm.ffclrp.usp.br/lssb/geas/). The GEAS Repository provides not only access to all available services, but also to detailed documentation about these services. The organization of the GEAS Repository was based on the DAVID Web Service repository [13], which represents a SOAP-based service repository that can be used by bioinformaticians to programmatically interact with DAVID in order to automate user tasks.

The documentation about each service in the GEAS repository is structured in six parts. The first part includes a general description about the service. The second part includes the service endpoint location (URL). The third part includes the description of the service itself (all supported operations). Each service operation is described in terms of its purpose, associated parameters and return type. The fourth part includes a graphical description specifying the service execution behavior from a user point of view. This specification consists of a workflow model in a graphical notation that represents the sequence of operation invocations (including alternate flows when relevant) expected by each service. The fifth part includes (sample) Java client codes that can be used to programmatically access the service functionality. We provide two separate sets of code files including at least one Java client code file that supports the invocation of each service operation and one Java sample analysis code file that illustrates how the provided Java client code can be used by implementing one possible sequence of operation invocation, as described by the expected service behaviour. This sample code can be modified to perform the intended analysis. We also include detailed instructions to help users use the sample code files. Finally, the sixth part includes the semantically annotated WSDL file defined for the service. The GEXPO and GEXPASO ontologies, which were used to semantically annotate all WSDL files, are also publicly available in our repository.

Table 1 summarizes all services available in the GEAS Repository, their goals and corresponding wrapped software tools.

**Table 1:** RESTful semantic web services available in the GEAS Repository.

Service	Service goal	Wrapped tool
MicroNorm	Preprocessing of one/two-color microarray data	affy and limma R packages
MicroOneDifferentialAnalysis	Differential analysis of one-color microarray data	R standard libraries
MicroTwoDifferentialAnalysis	Differential analysis of two-color microarray data	R standard libraries
RnaSeqDifferentialAnalysis	Differential analysis of RNA-Seq	DESeq2 R package
MicroHCluster	Hierarchical clustering of one/two-color microarray data	Cluster 3.0 tool
MicroKCluster	K-means clustering of one/two-color microarray data	Cluster 3.0 tool
MicroHClusterViewer	Visualization of hierarchically clustered microarray data	Java TreeView tool
EnrichmentAnalysis	Enrichment analysis of gene expression data	gProfileR R package
DAVID-REST	Enrichment analysis of gene expression data	SOAP-based DAVID web service
GeneSetEnrichmentAnalysis	Gene set enrichment analysis of gene expression data	gage R package
KeggPathwayViewer	Visualization of gene expression data rendered into KEGG pathways	pathview R package

### Integrated Analysis of Gene Expression Data

This section presents two integration scenarios in which a number of services developed in the context of this work are applied. The first scenario was devised to reproduce part of a study presented in [26] comprising the analysis of one-color microarray data. The second was devised to reproduce part of a study presented in [27] comprising the analysis of RNA-Seq data.

Each integration scenario is publicly available as a web-application at the GEAS Repository (see Sample Analysis Scenarios). Using these web-applications, it is possible to directly execute the integration scenarios in order to reproduce in real-time the results of our analysis. In addition, the source gene expression data files used in our integration scenarios as well as the intermediary results obtained from the execution of each analysis activity can be downloaded. Finally, all parameters used in our analysis are set as the default options for each analysis activity. However, it is possible to change them in order to explore other results.

#### Microarray Data Analysis

The study comprised the analysis of different sets of microarray data. Initially, gene expression profiles of bone marrow-derived multipotent Mesenchymal Stromal Cells (MSCs) isolated from healthy donors and Multiple Sclerosis (MS) patients before treatment with Autologous Hematopoietic Stem Cell Transplantation (AHSCT) were compared. Then, gene expression profiles of bone marrow-derived MSCs isolated from MS patients before and after treatment with AHSCT were also evaluated.

In order to reproduce each analysis, a number of activities were carried out in the following order: microarray data normalization, differential gene expression analysis, functional analysis and pathway visualization.

After defining this sequence of activities, we have selected different services to perform each defined activity. We have selected the *MicroNorm* and *MicroOneDifferentialAnalysis* services for normalization and differential analysis, respectively. For the functional analysis activity, we have initially selected the *EnrichmentAnalysis* service. However, the results obtained using this service were limited when compared to the results obtained using the *DAVID-REST* service due to differences in the statistical methods. Therefore, we have then selected the *DAVID-REST* service for functional analysis. Finally, we have selected the *KeggPathwayViewer* service for the visualization of KEGG pathways.

In our integration scenario, Agilent one-color microarray data were initially normalized using the *MicroNorm* service. Normalized microarray data was then submitted to the *MicroOneDifferentialAnalysis* service for the identification of differentially expressed genes. In order to facilitate the execution of the *MicroOneDifferentialAnalysis* service, normalized microarray data stored on a single multi-column dataset should be partitioned into two different datasets, each containing a distinct experimental condition. The resulting list of differentially expressed genes was then submitted to the *DAVID-REST* service for the identification of the most relevant KEGG biological pathways and Gene Ontology (GO) terms associated to the interest genes. Once again, in order to facilitate the execution of the *DAVID-REST* service, the list of differentially expressed genes should be partitioned into two different datasets, each representing up-regulated and down-regulated genes, respectively. Additionally, Agilent probe identifiers stored on these lists should be converted into respective Entrez gene identifiers [61].

The resulting enriched and normalized data were then submitted to the *KeggPathwayViewer* service for the visualization of gene expression data rendered into previous identified KEGG pathways. In order to facilitate the execution of the *KeggPathwayViewer* service, enriched data should be filtered in order to select only relevant KEGG pathways and associated genes. Additionally, normalized microarray data should be filtered in order to select only expression values related to the genes associated to each selected KEGG pathway.

**Fig 7:**
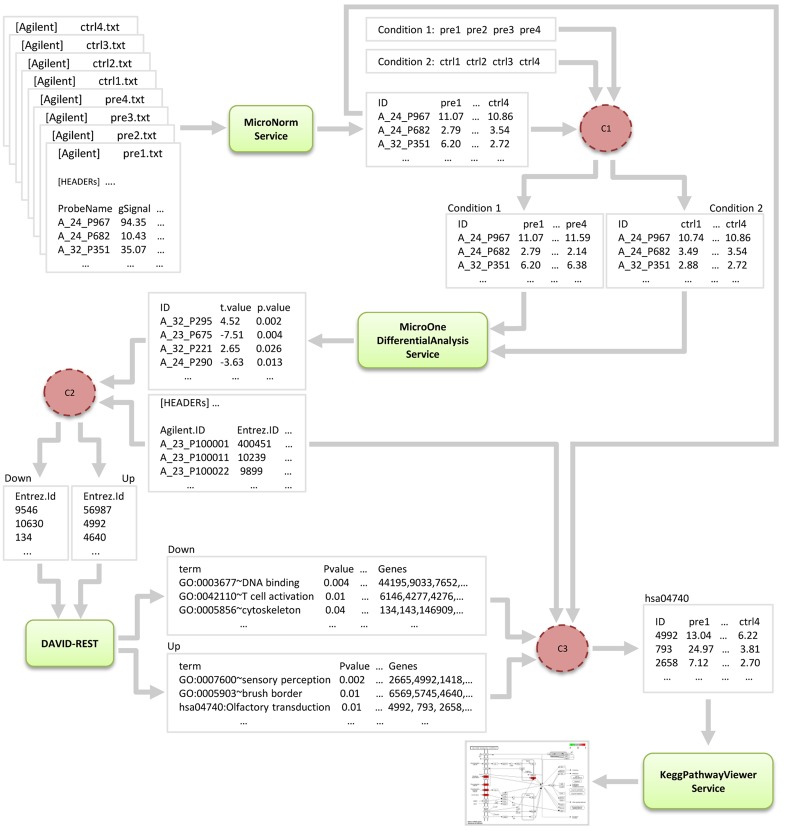
One-color microarray data analysis scenario service architecture. A rectangle represents a data source, while a rectangle with rounded corners represents a RESTful analysis service. A dotted circle represents a RESTful software connector. An one-way arrow represents a directed flow of data and/or control.

During the definition of this scenario, we have realized that interactions among services in our pipeline were not straightforward, and that some adaptation of the data to be exchanged would be required in order to guarantee service interoperability. Thus, in order to compose one service output with another service input we have defined a number of software connectors. A software connector is an architectural element used to accommodate different types of interactions among computational/data components of a software system through the definition of a set of rules that govern these interactions. In general, software connectors allow the transfer of data and/or control between different components of a software system. The identified connectors were initially developed as standalone applications using the methodology proposed in [55] and latter wrapped as RESTful web services using the methodology proposed in this work. The development and benefits of using semantic software connectors are discussed in [55]. Fig 7 shows the architecture of our analysis scenario with focus on the data flow.

Three software connectors were developed to integrate one-color microarray data to the *MicroOneDifferentialAnalysis*, *DAVID-REST* and *KeggPathwayViewer* services: connector *C1* partitions normalized microarray data produced by the *MicroNorm* service into two separate datasets to facilitate identification of differentially expressed genes by the *MicroOneDifferentialAnalysis* service; connector *C2* converts the list of gene identifiers produced by the *MicroOneDifferentialAnalysis* service and partitions this list into two separate datasets to facilitate enrichment analysis by the *DAVID-REST* service; and connector *C3* processes enriched data produced by the *DAVID-REST* service and normalized data produced by the *MicroNorm* service in order to facilitate visualization of KEGG pathways by the *KeggPathwayViewer* service.

Initially, different files used to store one-color microarray data, each representing a separate biological sample, were submitted to the *MicroNorm* service. This service was responsible for reading all these files and for producing a single (multi-column) file containing normalized data for all samples (each represented in a single column). This output file was then submitted to connector *C1*, which was responsible for reading this file and for producing two (multi-column) files containing normalized data partitioned according to two different experimental conditions: bone marrow-derived multipotent MSCs obtained from MS patients at pre-AHSCT and bone marrow-derived MSCs obtained from healthy donors. Connector *C1* also received two user-provided inputs specifying which columns represent each of the two experimental conditions, respectively.

After that, the two output files produced by connector *C1* were submitted to the *MicroOneDifferentialAnalysis* service. This service was responsible for selecting genes that had a greater than two-fold (user-defined) increase/decrease in expression between the two experimental conditions and for producing a list of differentially expressed genes. The resulting list was then submitted to connector *C2*, which was responsible for reading gene identifiers stored in this list, converting them into respective Entrez gene identifiers according to a user-provided mapping file, and for partitioning this list into two different datasets, each representing up-regulated and down-regulated genes, respectively. The two lists produced by connector *C2* were then submitted to the *DAVID-REST* service.

The *DAVID-REST* service was responsible for reading Entrez gene identifiers stored in the lists, and for identifying the most relevant KEGG pathways and GO terms associated to the interest genes. After that, resulting enriched data and normalized data were then submitted to connector *C3*, which was responsible for selecting only relevant KEGG pathways and associated genes on enriched data. For each selected KEGG pathway, connector *C3* was also responsible for filtering normalized data in order to select only expression values related to the associated genes, and for producing a multi-column file containing these values for all samples (each represented in a single column). Connector *C3* also received a user-provided mapping file to enable filtering of normalized data containing Agilent probe identifiers according to Entrez gene identifiers specified in enriched data. Finally, each file produced by connector *C3* for each relevant KEGG pathway was submitted to the *KeggPathwayViewer* service, which was responsible for producing a corresponding image that shows gene expression data rendered into the KEGG pathway graph.

The differential analysis of the transcriptional profiles of pre-AHSCT MS patients and healthy controls using the *MicroOneDifferentialAnalysis* service identified 488 differentially expressed genes (*p* < 0.05; fold-change ≥ 2.0), comprising 234 down-regulated and 254 up-regulated genes. Enrichment analysis performed by the *DAVID-REST* service revealed that most down-regulated genes were included in the following GO categories: *lymphocyte activation*, *T cell activation*, *leukocyte activation*, *gamma-delta T cell activation* and *activation of immune response*. Up-regulated genes were included in the following GO categories: *cell surface receptor linked signal transduction*, *neurological system process*, *G-protein coupled receptor protein signaling pathway* and *regulation of tissue remodeling*. Additionally, this analysis revealed up-regulated genes involved into two different KEGG pathways, including the *Neuroactive ligand-receptor interaction* pathway. These pathways were then visualized using the *KeggPathwayViewer* service.

The differential analysis of the transcriptional profiles of pre-AHSCT and post-AHSCT MS patients using the *MicroOneDifferentialAnalysis* service identified 75 differentially expressed genes (*p* < 0.05; fold-change ≥ 2.0), all up-regulated in post-transplantation MSCs. Enrichment analysis performed by the *DAVID-REST* service revealed that most genes were included in the following GO categories: *protein-DNA complex assembly*, *cellular macromolecular complex assembly*, *cellular macromolecular complex subunit organization*, *translation* and *translation elongation*. Additionally, this analysis also revealed a set of genes involved in the *Ribosome* KEGG pathway, which were then visualized using the *KeggPathwayViewer* service.

#### RNA-Seq Data Analysis

This study comprised the analysis of different sets of RNA-Seq data. Initially, gene expression profiles of BRG1 knockdown melanoma cells and control melanoma cells were compared. Then, gene expression profiles of MITF knockdown melanoma cells and control melanoma cells were also evaluated. Finally, the results obtained for each dataset were compared.

In order to reproduce the study, two activities were carried out in the following order: identification of differentially expressed genes and functional analysis. The *RnaSeqDifferentialAnalysis* and *DAVID-REST* services were selected for differential and functional analysis, respectively.

In this integration scenario, the two Illumina HiSeq 2500 RNA-Seq datasets comprising BRG1-control and MITF-control data were initially submitted to the *RnaSeqDifferentialAnalysis* service for the identification of differentially expressed genes. In order to facilitate the execution of the *DAVID-REST* service, each resulting list of differentially expressed genes should be partitioned into two different datasets, each representing up-regulated and down-regulated genes, respectively. The resulting datasets of up-regulated genes for BRG1-control and MITF-control were then compared and the commonly up-regulated genes were extracted to a single dataset. Similarly, the resulting datasets of down-regulated genes were compared and the commonly down-regulated genes were extracted to another dataset. Finally, the resulting datasets comprising commonly up-regulated and down-regulated genes were submitted to the *DAVID-REST* service for the identification of the most relevant Gene Ontology (GO) terms associated to the interest genes contained in each dataset.

One software connector was developed to integrate RNA-Seq data between the *RnaSeqDifferentialAnalysis* and *DAVID-REST* services: connector *C4* partitions each list of gene identifiers produced by the *RnaSeqDifferentialAnalysis* into two separate datasets and extracts commonly up-regulated and down-regulated genes into two other separate datasets to facilitate enrichment analysis by the *DAVID-REST* service.

Initially, different files used to store RNA-Seq data, each representing a separate biological sample, were submitted to the *RnaSeqDifferentialAnalysis* service. This service was responsible for reading all these files and for selecting genes that had a greater than two-fold (user-defined) increase/decrease in expression between the two experimental conditions and for producing a list of differentially expressed genes. This first step was separately performed for both BRG1-control and MITF-control datasets. After that, the two output files produced by *RnaSeqDifferentialAnalysis* service were submitted to connector *C4*. This connector was responsible for reading the files and for partitioning each file into two different datasets, each representing up-regulated and down-regulated genes, respectively. Connector *C4* was also responsible for extracting commonly up-regulated and down-regulated genes between BRG1-control and MITF-control. The resulting datasets comprising commonly up-regulated and down-regulated genes were finally submitted to the *DAVID-REST* service, which was responsible for reading gene identifiers stored in the datasets, and for identifying the most relevant GO terms associated to the interest genes.

The differential analysis of the transcriptional profiles of BRG1 knockdown and control cells using the *RnaSeqDifferentialAnalysis* service revealed 8762 differentially expressed genes (*p*.*adj* < 0.05; fold-change ≥ 2.0), comprising 3924 down-regulated and 4838 up-regulated genes. The differential analysis of the transcriptional profiles of MITF knockdown and control cells revealed 1266 differentially expressed genes (*p*.*adj* < 0.05; fold-change ≥ 2.0), comprising 532 down-regulated and 734 up-regulated genes. Around 90% of genes encountered by differential analysis using the *RnaSeqDifferentialAnalysis* service were also revealed in the original study [27]. Additionally, similar results were obtained regarding commonly expressed genes in BRG1-control and MITF-control datasets: 327 (61%) genes down-regulated by shMITF presented loss of expression upon shBRG1 and 272 (37%) commonly up-regulated genes were revealed.

Similar results were also obtained in the enrichment analyses performed by the *DAVID-REST* service. The analysis of commonly down-regulated genes revealed genes included in the following GO categories: *neuronal synaptic plasticity*, *intracellular signalling cascade*, *transcription regulation*, *cell division*, *mitosis* and *organelle fission*. The analysis of commonly up-regulated genes revealed genes included in the following GO categories: *cell adhesion*, *angiogenesis*, *cell projection organization*, *regulation of cell proliferation*, *cell morphogenesis* and *cell migration*. These results are consistent with the results obtained from the original study.

## Discussion

We have proposed a systematic methodology for the development of RESTful semantic web services for gene expression analysis. Our methodology supports not only the development of a RESTful web service from an existing application but also the semantic annotation of the corresponding service description using SAWSDL. The methodology was then applied to the development of a representative set of services for gene expression analysis, which have been applied in two scenarios.

In our opinion, the creation of semantic web services in the biomedical domain has not yet been addressed extensively enough. The SADI project [24] defines an architecture pattern specification to support the creation and integration of services in this domain. However, SADI-compliant services are created according to a non-standard service architecture specification and, unlike our approach, relies on the use of RDF and OWL (version 1) languages for data representation and semantic modelling.

Domain-independent approaches have been defined for the development of RESTful web services from existing tools. For example, Liu *et al.* [62] propose a common process for re-engineering data and entity-driven systems with RESTful web services. In this process, candidate web services are identified by analysing the legacy systems to be made accessible. Mapping rules are then defined for the generation of URIs, and finally the generated URIs are refined and split to represent the RESTful web services. Laitkorpi *et al.* [63] introduce a model-driven process for the development of RESTful web services from functional specifications originated either from new services or from legacy services requirements. In this work, the process phases and model transformations required to transform these functional specifications into WADL service descriptions are specified. However, code generation is out of scope of their work. Finally, Oldevik *et al.* [25] present a model-driven approach for the specification and generation of SOAP-based or RESTful web services from existing software applications. In this work, the authors define a UML profile and a set of code generators for generating service wrappers.

Other works focus on the semantic annotation of web services using SAWSDL. For example, Belouadha *et al.* [64] present a model-driven approach for the development and composition of SAWSDL semantic web services. In this approach, web services are modeled according to a UML profile and transformation rules are specified to map these created models to SAWSDL service descriptions. Zhang *et al.* [65] propose a semi-automatic method for the semantic annotation of XML Schema elements contained in WSDL documents using SAWSDL. According to this method, XML Schema elements are mapped onto OWL elements of a schematic ontology based on defined transformation rules. Once a schematic ontology is constructed, they use a specific algorithm for evaluating semantic affinity between concepts of this ontology and concepts contained in domain ontologies. The terms from domain ontologies with highest semantic affinity values are used to annotate XML Schema elements contained in a WSDL document using the SAWSDL attribute *modelReference*. Lastly, Gordon *et al.* [66] present a real-world application of the SAWSDL approach in the context of the BioMOBY project, which aims at providing interoperability between biological data. In this work, the authors present a software system that allows the creation of Moby-compliant semantic web services by simply adding SAWSDL annotations to existing WSDL documents.

In contrast to both sets of approaches that independently tackle either the development of wrappers for creating RESTful web services from legacy software applications or the semantic annotation of these services, our work provides the first comprehensive methodology that addresses both service development and semantic annotation. Still, the development of RESTful semantic web services could be achieved using two independently defined approaches. However, the combined use of such approaches is likely to require some adaptations in order to make them compatible. Furthermore, many model-driven and usually domain-specific approaches have been reported in the literature, and, to the best of our knowledge, no suitable approach has already been defined for the gene expression analysis domain.

Since different standalone tools have already been developed for the execution of one or more gene expression analysis activities, the primary focus of our methodology was to facilitate the development of a web service based on an existing standalone tool and then the semantic annotation of this service. Our methodology does not address the development of a RESTful web service from scratch. Nevertheless, in order to address the development of such services, it would only be necessary to modify (extend) the proposed service implementation activity to include a suitable set of software development activities to cope with the whole software development cycle. The remaining activities of our methodology would likely to be kept unmodified.

In recent years, the explicit use of semantics has been highlighted as crucial to enable automatic service composition. In the context of this work, we have simply used the semantic annotations assigned to the developed services implicitly in the development of our integrated analysis scenario. These semantic annotations were used to reason about service functionality, inputs and outputs, thus assisting the manual selection of the most suitable services at each step of our analysis scenario. However, we believe our work contributes to the development of a complete solution for (semi) automatic service composition. We have proposed a methodology for the development of semantic web services, applied this methodology to the development of a representative set of services and validated the use of these services through a real case study. Additionally, even though no complete solution to automatic service composition is available, a biologist can still benefit from the provided semantic annotations to properly select/discover a suitable service for performing a task at hand.

We have demonstrated the validity and usefulness of the developed RESTful semantic web services through the creation of two integrated analysis scenarios to analyze real sets of gene expression data. In the first scenario, by using the available set of services, we were able to reproduce a specific set of results reported in [26]. The results of the analysis of transcriptional profiles (pre-AHSCT versus control and pre-AHSCT versus post-AHSCT) included 44.5% and 92.6% of the differentially expressed genes reported in the original study, respectively. These differences do not indicate a limitation of our services, since we have used a distinct set of tools and statistical methods than those used in the original study for data normalization and differential analysis. Most importantly, the subsequent enrichment analysis carried out on resulting data partially revealed a different set of relevant GO categories and KEGG pathways than those found in the original study, including *activation of immune response*, *tissue homeostasis*, *translational elongation*, *translation*, *chromatin* and *ribosome*. The biological relevancy of these findings is yet to be determined in additional studies, but this falls outside the scope of this work.

In the second scenario, we were able to reproduce a specific set of results reported in [27]. The results of the analysis of transcriptional profiles (BRG1 knockdown versus control and MITF knockdown versus control) included 91.6% and 88.8% of the differentially expressed genes reported in the original study, respectively. The comparative analysis of transcriptional profiles (BRG1-control versus MITF-control) included 86.9% and 93.6% of commonly down-regulated and up-regulated genes reported in the original study, respectively. The subsequent enrichment analysis carried out on resulting data revealed a very similar set of relevant GO categories than those found in the original study, including all categories that were considered most relevant.

Finally, the proposed methodology entails a fair amount of technical details, which make it unlikely to be grasped by a biologist. However, bioinformaticians and associated IT personnel can greatly benefit from our systematic approach to the development of RESTful semantic web services for gene expression analysis, since they represent the target users of our methodology. Similarly to our methodology, the GEAS repository target users are bioinformaticians/IT personnel. In this sense, the available set of services can be used for the creation of integrated analysis environments suitable to the needs of specific end users.

To the best of our knowledge, our work provides the first methodology for the development and semantic annotation of RESTful web services from existing software tools in the gene expression analysis domain. We provide not only a methodology for the creation of such services, but also two support ontologies that can be possibly used in the semantic annotation of other services pertaining to this domain. In contrast to other existing approaches, our methodology provides a generic solution that does not constrain the type and/or structure of services being developed. Therefore, our methodology provides a flexible solution for service development and reuse through the adaptation of different types of analysis tools.

Additionally, most existing approaches lack concrete guidelines concerning the technical issues of the implementation and semantic annotation of RESTful web services, which are covered in our methodology. We have also discussed the application of our methodology in the development of a representative web service for gene expression data rendering into KEGG pathways in sufficient detail so that it can be reproduced or applied to other tools. This detailed description was also intended to reduce the learning time of our methodology, thus facilitating its application.

Service-oriented architecture has been increasingly used in the development of computer systems in a wide range of knowledge domains. The benefits of service-based development include faster and cheaper development since services can be reused to create new services, and increased reliability due to the reuse of tested solutions. In particular, our work encourages the development of new services and subsequent reuse of such services for the creation of integrated solutions for gene expression analysis. Although our methodology has been defined for this particular domain, it can be easily extended to cope with other biological domains simply by replacing our domain ontology by an ontology suitable for the new domain and by adapting the service ontology, if necessary.

The gene expression analysis scenarios proposed in this paper were implemented by manually composing a number of available services and making implicit use of the semantics assigned to the developed services. However, (semi) automatic service composition requires the explicit use of semantics throughout the service composition life-cycle, including requirement specification, service discovery, service composition and service execution. Therefore, as future research we intend to investigate the (semi) automatic composition of gene expression analysis services, making explicit use of the semantic annotations we assigned to our developed services.

## Conclusions

Semantic web services have been increasingly used in the biomedical domain. Not only new services have been created from scratch, but also existing software tools have been adapted to be accessed through semantic web services. In this work, we have defined an integrated methodology that addresses both the implementation and semantic annotation of RESTful web services created from existing analysis tools. Our methodology provides concrete guidelines and technical details, thus facilitating the systematic adaptation of analysis tools. Moreover, we believe our methodology fosters the development and reuse of semantic web services for the creation of integrated solutions for gene expression analysis.

Our methodology has been applied to the development of a suite of services for the analysis of one/two-color microarray data and RNA-Seq data. This suite of services provides support for data normalization, differential analysis, hierarchical and k-means clustering, clustering visualization, enrichment analysis and pathway visualization. A number of these services were successfully combined to create two semantically integrated analysis scenarios aiming at reproducing parts of different studies documented in the literature.

Future research includes the development of new services to support new analysis activities, such as gene network inference. Finally, in the future we intend to work towards the definition of an approach for the (semi) automatic composition of gene expression analysis services. This approach should rely on the use of semantic software connectors to facilitate the interconnection of different services. Ultimately, we intend to build an environment to support the (partly) automated composition of RESTful semantic web services in the gene expression domain and to assist the biologist when creating suitable analysis solutions.
